# A Method for Combining RNAscope In Situ Hybridization with Immunohistochemistry in Thick Free-Floating Brain Sections and Primary Neuronal Cultures

**DOI:** 10.1371/journal.pone.0120120

**Published:** 2015-03-20

**Authors:** Tessa M. Grabinski, Andrew Kneynsberg, Fredric P. Manfredsson, Nicholas M. Kanaan

**Affiliations:** Department of Translational Science and Molecular Medicine, College of Human Medicine, Michigan State University, Grand Rapids, MI, United States of America; University Hospital Basel, SWITZERLAND

## Abstract

In situ hybridization (ISH) is an extremely useful tool for localizing gene expression and changes in expression to specific cell populations in tissue samples across numerous research fields. Typically, a research group will put forth significant effort to design, generate, validate and then utilize *in situ* probes in thin or ultrathin paraffin embedded tissue sections. While combining ISH and IHC is an established technique, the combination of RNAscope ISH, a commercially available ISH assay with single transcript sensitivity, and IHC in thick free-floating tissue sections has not been described. Here, we provide a protocol that combines RNAscope ISH with IHC in thick free-floating tissue sections from the brain and allows simultaneous co-localization of genes and proteins in individual cells. This approach works well with a number of ISH probes (e.g. small proline-rich repeat 1a, βIII-tubulin, tau, and β-actin) and IHC antibody stains (e.g. tyrosine hydroxylase, βIII-tubulin, NeuN, and glial fibrillary acidic protein) in rat brain sections. In addition, we provide examples of combining ISH-IHC dual staining in primary neuron cultures and double-ISH labeling in thick free-floating tissue sections from the brain. Finally, we highlight the ability of RNAscope to detect ectopic DNA in neurons transduced with viral vectors. RNAscope ISH is a commercially available technology that utilizes a branched or “tree” *in situ* method to obtain ultrasensitive, single transcript detection. Immunohistochemistry is a tried and true method for identifying specific protein in cell populations. The combination of a sensitive and versatile oligonucleotide detection method with an established and versatile protein assay is a significant advancement in studies using free-floating tissue sections.

## Introduction

In situ hybridization (ISH) is an extremely powerful research tool that can provide a mechanism of identifying gene expression changes in specific cell population, and with the appropriate methods the sensitivity is remarkable. Over the years, a number of large databases have been generated that provide ISH-related data on many genes from multiple tissue sources (e.g. the Allen Brain Atlas, Eurexpress, the Edinburgh Mouse Atlas Gene Expression Database, and the GenePaint database). There have been significant advancements with the ISH method since the first description of ISH by Gall and Pardue (1969)[1]. Initially, probes containing radiolabeled nucleotides were used to detect mRNA transcripts. However, many of the more recent variations use alternative forms of detection. This includes probes labeled with digoxigenin, biotin, enzymes (i.e. horseradish peroxidase, alkaline phosphatase) or fluorophores (used in fluorescent ISH methods). These methods provide distinct advantages over radiolabel methods by providing more sensitive detection, cell localizability and are unburdened by radioactivity concerns.

The sensitivity of ISH methods has remained a concern, and subsequently methods aimed at enhancing ISH sensitivity continue to be pursued (reviewed in [2–6]. The use of transcript amplification using *in situ* PCR or *in situ* transcription have been developed. Despite the relatively straightforward conceptual underpinnings of these methods, the practical application of *in situ* PCR has not yielded satisfactory results. The amplification of signal detection using biotinylated tyramine has proven useful in significantly enhancing sensitivity [7], and some forms of FISH hold promise in enhancing resolution [8]).

Among some of the newest ISH methods is RNAscope, a commercially available branched (or “tree”) ISH assay [9]. The ability to detect single mRNA transcripts using standard microscopy techniques is the biggest advantage of this method [9–11]. For example, Wang and colleagues [9] demonstrated the ability of RNAscope to detect single transcripts in three ways. First, a probe set was used to identify the human epidermal growth factor receptor 2 (HER2) gene in DNA of HeLa. In HeLa cells, two fluorescent puncta were observed, which is consistent with the HER2 gene diploidy in this cell line. Secondly, they found that the number of HER2 mRNA puncta per cell (manually counted) matched very well to the number of mRNA transcripts per cell (as measured by QuantiGene 2.0) in HeLa cells. Thirdly, they measured the intensity of fluorescent puncta from HER2 DNA labeling to the intensity of HER2 mRNA puncta and found that fluorescent intensities were similar suggesting the mRNA puncta represent single transcripts. Thus, RNAscope is a useful method for labeling oligonucleotide molecules with high sensitivity.

The use of immunolabeling techniques is commonplace among many fields of biological research to identify proteins in cells and tissue sections. A prominent form of immunolabeling that has been used for many decades is immunohistochemistry (IHC) [12]. These techniques utilize chromogenic enzyme substrates that are deposited as variously colored products. IHC techniques are powerful means to identify specific cell populations expressing specific proteins (i.e. the antigen). Typically, the target protein is detected by labeling it with a specific primary antibody, which is then labeled with a secondary antibody raised against the primary antibody host species immunoglobulin. Often, the secondary antibody is tagged with biotin to facilitate detection of the antigen-primary-secondary complex with avid-biotin systems that introduce the enzyme (peroxidase or phosphatase substrates are common) for converting the chromogen into the colored product. Various iterations of IHC (and immunolabeling in general) have been developed over time and this technique is well accepted among the biological sciences [12].

The combination of mRNA analysis via ISH and protein analysis via IHC in the same section is an extremely powerful technique that allows one to answer important biological questions that are difficult to address using other methods. Here, we describe a method for combining ISH (i.e. RNAscope technology) with IHC in thick free-floating tissue sections from the brain. This method of ISH is exceptionally sensitive (i.e. single transcript sensitivity) and provides exquisite cellular localization, which can be coupled with IHC to identify specific cell populations and/or cells expressing certain proteins. We demonstrate the versatility of this approach using a number of ISH-IHC combinations, including IHC markers for both neurons and glial cells. These methods are applicable to tissue sections cut at various thicknesses (20 and 40μm), archived tissue resources, adherent cell cultures (e.g. primary neurons), detecting mRNA and ectopic DNA, and for multi-plexing ISH in the same section. Moreover, the signal from the ISH is semi-quantitative using this method. Combining ultrasensitive, semi-quantitative ISH to identify mRNA/DNA with standard IHC to identify specific cell populations in thick free-floating tissue sections will prove to be an important technique in the neurosciences, as well as a number of research fields.

## Materials and Methods

### Animals

Adult male Sprague Dawley rats were used for all experiments. The goal of this manuscript is to provide a detailed account of the ISH/IHC method, but the primary gene of interest (i.e. small proline-rich repeat 1a, Sprr1a) used to develop this method is induced following injury in neurons [13,14]. Thus, dopaminergic neurons in the nigrostriatal system were lesioned using 6-hydroxydopamine (6-OHDA) as described previously [15]. The animals were sacrificed 7–8 days after 6-OHDA administration (a time when significant Sprr1a upregulation occurs). For detection of ectopic DNA the animals were injected intracerebrally (i.e. into the striatum) with recombinant adeno-associated virus (rAAV) engineered to express GFP as described [16]. The animals were sacrificed one month following the delivery of rAAVs. Timed pregnant female Sprague-Dawley rats (embryonic day 18, E18) were used for primary hippocampal neuron cultures (see below). All animal studies were performed in accordance with standard regulations and were approved by the Michigan State University Institutional Animal Care and Use and Committee.

### Tissue processing

Animals were transcardially perfused with ∼200ml of 0.9% saline containing heparin (10,000 U/L), followed by ∼200ml phosphate buffered 4% paraformaldehyde. The brains were post-fixed in 4% paraformaldehyde for 18–24 hours. After post-fixation, the brains were equilibrated to 15% sucrose, followed by 30% sucrose and then cut into either 20μm or 40μm thick sections on a freezing, sliding stage microtome. Sections were stored in cryoprotectant until processed for RNAscope/IHC.

Although not used here, immersion fixation is an alternative method of tissue fixation that can be successfully used for RNAscope ISH and IHC analyses (Kanaan personal communication). In this approach, the animals are perfused with saline, but not paraformaldehyde. Instead, the brain is immersed in 4% paraformaldehyde for 48–72 hours. This method is advantageous for obtaining both fixed and fresh tissue from the same animal allowing one half of the brain to be used for fresh tissue analyses (e.g. Western blots) while the other can be used for fixed tissue analyses (e.g. IHC and ISH) [17].

### Primary Neuron Growth and Processing

Primary neurons were generated from dissected E18 rat hippocampi following a similar procedure as described previously [18] with the following modifications. The tissue pieces were incubated in 0.125% trypsin for 15 min at 37°C prior to dissociation. To obtain a single cell suspension, trituration was performed by gently passing the tissue through a 3ml syringe with a 14g needle 30 times, 15g needle 30 times, 17g needle 20 times, 18g needle 20 times and finally a 21g needle 15 times. Cells were plated on poly-D-lysine coated chamber slides (Millipore, PEZGS0816) at a density of 40,000 cells/cm^2^ and grown in neurobasal medium (Gibco, 21103–049) supplemented with L-glutamine (Gibco, 25030–081), fungizone (Gibco, 15290–018), gentamicin (Gibco, 15750–060) and B27 (Gibco, 17504–044). After 7 days, the cells were fixed with 4% paraformaldehyde for 30 min. Following fixation the cells were rinsed 3 times in TBS and then processed for RNAscope/IHC as described below.

### RNAscope ISH Procedure

RNAscope is commercially available from Advanced Cell Diagnostics (ACD) [9]. ACD has a large library of validated probes (for mouse, human and rat genes) and ACD will design custom probes per customer requests. Typically, the standard probes contain 20 ZZ probe pairs (50bp/pair) covering ∼1000bp, but the probes can be longer or shorter depending on the desired target region. Here, we used probes against rat small proline-rich repeat 1a (Sprr1a, NM_021864.1, bp3–801, ACD# 407411), rat tau (NM_017212.2, bp281–1281, ACD# 409001-C2), rat βIII-tubulin (NM_139254.2, bp484–1412, ACD# 409061), rat β-actin (NM_031144.3, bp212–1177, ACD# 409051) and the chicken β-actin portion of a chicken β-actin/cytomegalovirus promoter in the rAAV genome DNA (ACD# 408321). Here, we describe protocols for either 20μm sections, 40μm sections or primary neuron cultures (see **S1 Protocol** for a detailed step-by-step laboratory protocol).

The RNAscope assay comes in a number of versions and the target probes have to be designed for the appropriate assay. The single-plex RNAscope assay utilizes a probe set against one target molecule and the probes are referred to as channel 1 probes. These probes can be used in the single-plex assay where they are detected with DAB (a peroxidase substrate), in the two-plex assay where they are detected with the blue-green chromogen (a peroxidase substrate), or in the multi-plex assay where they are detected with FITC. The two-plex assay incorporates a second probe set against a different target molecule and these probes are referred to as channel 2 probes. These probes can be used in the two-plex assay, but cannot be used in the single-plex assay. Two-plex probes are detected with the red chromogen (an alkaline phosphatase substrate) and with Cy3 fluorophore in the multi-plex assay. Finally, the multi-plex assay uses additional probe sets called channel 3 and 4 probes that are detected with Cy5 and Cy7 fluorophores (channel 3 and 4 probes cannot be used in the single- or two-plex assays). Thus, each probe set must be designed to work in the appropriate assay. Here, we use a number of channel 1 probes against different target molecules in the single-plex assay and provide an example of the two-plex assay.

#### 20μm free-floating tissue section protocol

Sections were removed from cryoprotectant and washed four times in tris buffered saline (TBS, 50mM Tris, 150mM NaCl, pH7.4) for 10 min each. The sections were incubated in Pretreatment 1 at room temperature (RT) until bubbling ceased (45–60 min). Sections were rinsed four times in 0.5xTBS for 1min each. Floating sections were then moved into 0.5xTBS and mounted (one section per slide is recommended) directly onto VistaVision Histobond slides (VWR, 16004–406) using the following procedure (Thermo UltraStik slides may increase adhesion with thicker sections, Fisher 3039–002). The sections were positioned and flattened on the slide (care should be taken to ensure there are no folds/overlaps in the section). Once the tissue begins to dry (∼30–60s), the bristles of a paintbrush were wetted and flattened between paper towels to fan out the bristles (**S1 Fig.)**. The tip of the flattened bristles was used to very carefully and gently flatten tissue. It is imperative that there are no wrinkles, no bulging areas and/or no bubbles under the tissue because this will significantly reduce the quality of the final results. If the tissue is not flat against the slide, the tissue can start to come off the slide during the procedure and/or increased tissue coloration may occur during the development process. Once mounted, the sections were dried onto the slides (either RT or 60°C) and then residual salts were removed by rapidly dipping the slides in H_2_O. Once the slides were clear of salt residue, they were dried overnight at 60°C on a slide warmer. The following day, the sections were incubated in a boiling (99–104°C) solution of 1X Pretreatment 2 for 5–10 min. The slides were washed in H_2_O two times for 1min each and then dried at RT. Once dry, the slides were dipped in 100% ethanol (EtOH) and air-dried before creating a hydrophobic barrier around the tissue using a Pap Pen (RPI, 195506). The barrier was allowed to completely dry at RT before proceeding with Pretreatment 3 (see below).

#### 40μm free-floating section protocol

The sections were removed from cryoprotectant and washed four times in TBS for 10 min each. Sections were incubated in Pretreatment 1 at RT until the bubbling ceased (45–60 min). The sections were washed four times in TBS for 1 min each. Floating sections were then placed in 1.5ml tubes containing 1X Pretreatment 2 pre-heated to 99–100°C and incubated for 10 min. Sections were *immediately* removed from Pretreatment 2 and placed in 0.5xTBS for mounting directly onto Histobond slides (one section per slide is recommended) using the same procedure outlined above. Again, the final quality of the staining is significantly affected if the sections are not properly mounted, cleaned of residual salts, and dried prior to moving to the next step. The sections were dried at 60°C overnight. The following day, sections were dipped in 100% EtOH and air-dried before creating a hydrophobic barrier around the tissue using a Pap Pen. Once the barrier was completely dried, Pretreatment 3 was carried out as described below.

#### Pretreatment 3

From this point, 20μm and 40μm sections were processed identically. Sections were incubated in Pretreatment 3 (∼2–3 drops/per section), taking care to completely cover the tissue. Then, the slides were incubated at 40°C for 15 min in the EZ Hybridization oven (ACD, 310010) using the humidity control tray (ACD, 310012) and slide rack (ACD, 310014) before washing four times in H_2_O for 1min each. All remaining incubation steps were done using this oven and tray system unless otherwise noted.

#### Probe Incubation for Single-Plex Probes

Probes were designed by ACD according to the specific mRNA/DNA target of interest and were ordered as single-plex probes for detection of one target oligonucleotide (see above for details). Tissue sections were incubated in desired probe (∼2–3 drops/section) for 2 hours at 40°C. The slides were washed four times in 1x wash buffer (ACD, 310091) for 1 min each.

#### Amp 1–6 for Single-Plex Probes

Amplification and detection steps were performed using the RNAscope 2.0 HD Detection Kit reagents (ACD, 320497) for single-plex probes. Sections were incubated with Amp1 for 30 min at 40°C and then washed four times in wash buffer for 1 min each. Amp2 was incubated on the sections for 15 min at 40°C, followed by four washes in wash buffer. Sections were incubated in Amp3 for 30 min at 40°C and washed four times in wash buffer for 1 min each, followed by incubation of Amp4 for 15 min at 40°C. Slides were washed four times in wash buffer for 1 min each. Slides were incubated with Amp5 for 30min at RT using the HybEZ humidity control tray and slide rack to maintain humidity. The slides were washed four times in 1x wash buffer for 1 min each and incubated in Amp6 for 15 min at RT before washing four times in wash buffer for 1 min each.

#### Detection for Single-Plex Probes

ISH signal was detected by mixing equal parts of diaminobenzidine (DAB)-A solution to DAB-B solution and incubating sections with this solution for 10 min at RT. Slides were washed in H_2_O two times for 2 min to stop the DAB reaction. Detection can be monitored under a microscope and stopped when desired signal is achieved. Slides were then processed for immunohistochemistry as described below. Alternatively, sections can be counterstained with hematoxylin QS (Vector Labs, H-3404) per the manufacturer’s instructions.

#### Detection of two-plex ISH in free-floating tissue sections

The detection of two ISH signals in thick free-floating tissue sections was performed following the same steps as above with the following exceptions (all incubation times remain the same unless otherwise noted). One of the probes used for the two-plex approach is the same probe that is used in single-plex approach (a “channel 1” probe), however, the second probe has to be specifically designed by ACD for use in the two-plex approach (a “channel 2” probe). The channel 2 probe was diluted at 1:50 in the channel 1 probe solution prior to application on the tissue sections. Amplification and detection steps were done using the RNAscope 2-Plex Detection Kit (ACD 320701). For amplification step 4, the Amp4B component was diluted 1:50 into the appropriate volume of Amp4A component (per the kit instructions). Detection of the red signal was achieved by diluting component Red-B 1:60 in component Red-A and incubating on the tissue for 30 minutes at room temperature. Slides were washed two times in water for 1 minute each to stop the chromogen reaction. Detection of the green signal was achieved by diluting component Green-B 1:50 in component Green-A and incubating for 10 minutes at room temperature (produces a blue-green colored product). Slides were washed four times in water for 1minute each to stop the reaction. Slides were then dried at room temperature and then cleared directly in xylenes before coverslipping with EcoMount. The slides should not be dehydrated through ethanol because the red chromogen is negatively affected and the slides should not be coverslipped using other mountants because the blue-green chromogen is soluble in other toluene-based products.

#### RNAscope in cultured primary neurons

Cells were incubated in Pretreatment 1 solution at RT for 10 minutes and then rinsed 2 times in TBS. Next, the cells were treated with Pretreatment 3 (diluted 1:15 in TBS) at RT for 5 minutes. After Pretreatment 3, the cells were rinsed 2 times with TBS and then incubated in the probe solution as described above. All of the remaining steps were the same as described above. Note that the cells should not be allowed to dry out at anytime in the protocol. Once the ISH signal detection was complete, the neurons were processed for βIII-tubulin as described below.

### Immunohistochemistry (IHC) after ISH detection

All of the following incubation steps were performed using the humidity tray and slide rack from the EZ Hybridization oven to maintain humidity during incubations. Immediately following wash steps after the ISH detection (from above), the tissue was smoothed onto the surface of the slide using a flattened paintbrush as described earlier (**S1 Fig.)**. Slides were dried at 60°C for several minutes before proceeding with IHC. Alternatively, slides can be dried at 60°C overnight before continuing with IHC. Once dry, reapply the hydrophobic barrier and allow it to completely dry before proceeding. The slides were incubated in 1% H_2_O_2_ (made in TBS) for 10 min at RT to quench residual peroxidase activity from the ISH labeling. Slides were then washed in TBS four times for 4 min each. Next, the slides were incubated in blocking buffer consisting of 10% goat serum (serum from the host species of secondary antibody should be used) for 30 min at RT in the humidity control rack and slide tray. Next, the samples were incubated in primary antibody and incubated at RT overnight. The tissue sections were incubated in either rabbit anti-tyrosine hydroxylase (TH, a marker of dopamine neurons; Millipore, AB152, 1:5000), mouse anti-βIII-tubulin (Tuj1, 1:5000, a generous gift from A. Frankfurter [19]), rabbit anti-GFAP (Dako, Z0334, 1:20,000), or mouse anti-NeuN (Millipore, MAB377, 1:2000) antibodies diluted in TBS containing 2% goat serum. The primary neurons were labeled using the mouse anti-βIII tubulin antibody as above.

The following day, the slides were washed four times in TBS for 4 min each. Goat anti-rabbit biotinylated secondary antibody (Vector, BA-1000) or goat anti-mouse biotinylated secondary antibody (Vector, BA9200) were each diluted 1:500 in 2% goat serum and the samples were incubated for 2 hours at RT. Slides were washed four times in TBS for 4 min each. The sections were incubated in avidin-biotin complex solutions prepared according the manufacturer’s recommendations (Vector Labs PK-6100) for 2 hours at RT. Slides were washed four times in TBS for 4 min each. IHC signal was detected using the VectorSG peroxidase substrate, which produces a blue-grey colored product that provides good contrast with the DAB product from the ISH. VectorSG was prepared according to manufacturer’s recommendation (Vector Labs, SK-4700) and was monitored using a microscope until desired staining intensity was achieved (∼5–8 min). The reaction was stopped by washing in TBS four times for 1 min each. After the final rinse, the slides were dehydrated through graded EtOH (i.e. 50%, 70%, 95%, and 100%) for 1 min each. The tissue was cleared in xylenes and coverslipped using Cytoseal-60 mounting media.

### Image analysis

Images were acquired on a Nikon Eclipse E800 microscope system at 20x magnification. All microscope and camera settings (i.e. light level, exposure, gain, etc.) were identical for all images. Images were analyzed in ImageJ software (v1.45s) using the “color threshold” function for hue, saturation and brightness (HSB). To analyze the images, each image is opened in ImageJ, under the Image>Adjust window the color threshold function is selected and under the threshold window the Hue/Saturation/Brightness (HSB) indexing option and default threshold method are selected. The settings for hue were set at 0–128, the saturation settings were 0–255 and the brightness settings were 0–141. These setting provided the best distinction between the brown (ISH) and blue (IHC) signals, while detecting the most amount of ISH signal possible (pixels within the set threshold limits are illustrated in bright red). Importantly, end users will need to identify the settings that provide the best distinction between the brown ISH signal and the blue IHC signal because the staining intensities, staining patterns, and image acquisition parameters will influence the settings needed to achieve the best separation of ISH from IHC. After applying the color threshold settings, the “analyze particle” function (under the Analyze menu) was used with the pixel size (pixel^2^) set from 0-∞ and circularity set from 0–1.0 to include all objects. Measurement outputs included the count of objects, total area (pixels^2^), fraction of total area and average object size (pixels) within the threshold settings. Two different approaches were used to analyze ISH signal, one regional and the other for individual cells. First, entire 20x images were used for regional analyses by applying the methods above and obtaining the output measures. Four separate images in the intact and lesioned substantia nigra were used for each animal (n = 3) and data are represented as the sum of object counts, average total area, average fraction area, and average object size. Second, individual neurons containing 4 levels of ISH signal (i.e. none, low, moderate and high) were analyzed using the same parameters as outlined above. Ten neurons per ISH intensity category were analyzed and data are represented as above.

The combination of staining presented in cultured neurons utilized a channel 2 probe (i.e. detected with red chromogen—red puncta) and blue IHC chromogen. Using the HSB indexing method described above for the brown puncta/blue IHC combination does not work with the red puncta/blue IHC combination. Instead, this combination of staining is best distinguished using the YUV indexing option and default threshold method. Here, the Y setting was 0–135, the U setting was 0–255 and the V setting was 132–255 (pixels within the set threshold limits are illustrated in bright red). Similar results were achieved using the L*ab* indexing option and default threshold method, where L was set to 0–190, *a* was set to 129–255 and *b* was set to 0–255 (data not shown). Again, the threshold settings need to be empirically determined by the end user with the goal of settings that provide the most amount of ISH puncta detection and smallest amount of blue IHC detection. Here, we did not perform an experimental manipulation that warrants obtaining the outcome measures from thresholding, but this thresholding method can distinguish the red ISH signal from the blue IHC signal (see below).

### Statistical Analyses

All data were analyzed using Prism software (v5.0). For regional analyses, the intact and lesioned hemispheres were compared using a two-tailed paired t-test for each outcome measure and statistical significance was set at p<0.05. For individual neuron analyses, the data were compared using a repeated measure one-way ANOVA with significance set at p<0.05. Post-hoc comparisons were performed using the Newman-Keuls multiple comparison test.

### Ethics Statement

All national regulations and guidelines for the humane care and use of animals were followed and the animal procedures were approved by the Michigan State University IACUC. The work pertaining to deriving primary neurons from timed pregnant female rats was covered by MSU IACUC protocol # 09/13–202–00, the 6-OHDA animal model work was covered by protocol # 06/11–120–00, and the rAAV animal work was covered in protocol # 02/12–027–00. Isoflurane gas anesthetic was used for all surgical procedures and buprenorphine was used for post-operative pain relief. Sacrifice of animals was performed using an overdose amount of pentobarbital followed by exsanguination (i.e. intracardial perfusion).

## Results

### RNAscope combined with IHC in thick free-floating brain sections

**Fig 1:**
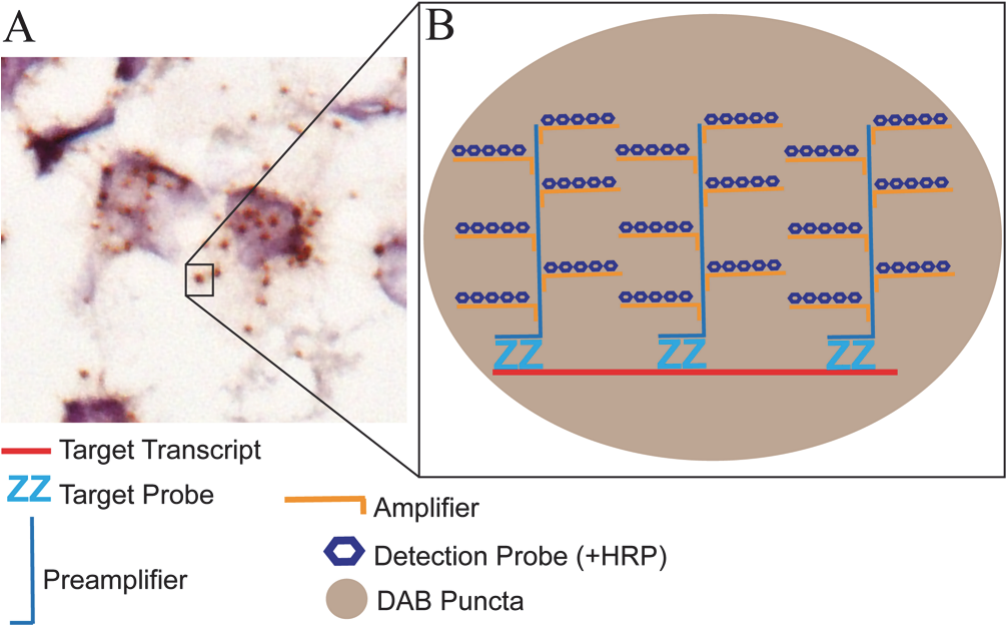
Illustration of RNAscope branched ISH method. RNAscope utilizes a branched ISH method that provides significant amplification and discrete detection of single transcripts. (A) RNAscope ISH produces puncta of signal that represent a single mRNA transcript. It is noteworthy, that at a certain level the amount of transcript can be elevated to a level where individual puncta are difficult to discern within single cells (see Figs. 2I and 5D as examples). (B) This level of sensitivity is achieved by initially hybridizing multiple tandem “Z” probes to the target transcript (these primers typically target 50bp long sequences and multiple sets of primers are made against the target molecule, ACD offers custom designed primers). Then a pre-amplifier probe is bound to the tandem Z probe sets, followed by the addition of multiple amplifier probes to the pre-amplifier probe. Finally, a number of detection probes that are conjugated to horseradish peroxidase (HRP) enzyme are added onto the amplifier probes and HRP substrate is added (i.e. diaminobenzidine, DAB). The resulting conversion of DAB to a brown colored reaction product by all of the HRPs bound the branched ISH complex produces discrete puncta. Alternatively, alkaline phosphatase (two-plex RNAscope) or fluorophores (multi-plex RNAscope) are conjugated to the detection probes for the appropriate version of the assay.

Our first goal was to combine a sensitive oligonucleotide detection method with IHC that would allow for the co-localization of gene expression in specific cell populations (marked by immunolabeling specific proteins). The branched or “tree” ISH method used by RNAscope technology is an extremely sensitive method for detecting oligonucleotides [10]. The resulting stain produces strong, discrete puncta, which were previously shown to represent the detection of individual mRNA or DNA molecules (**Fig. 1**) [9,11]. If a highly expressed transcript is detected the puncta can fill a large extent of the cytoplasm making individual puncta difficult to discriminate.

**Fig 2:**
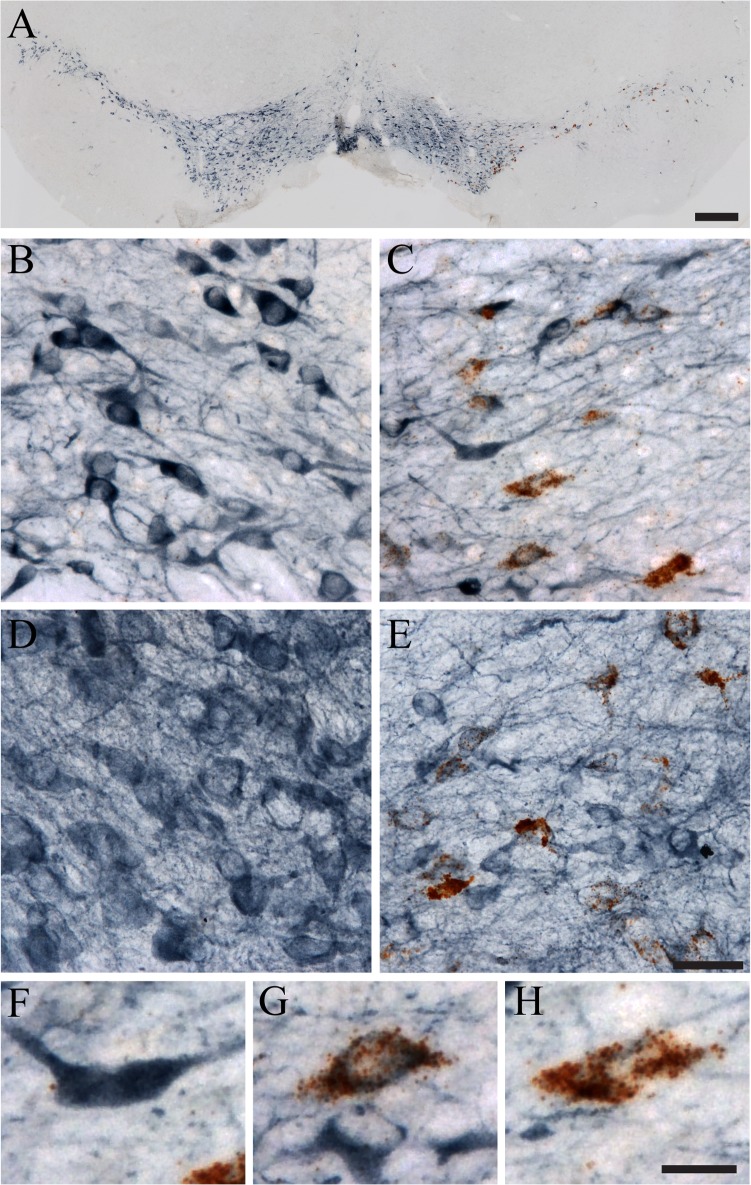
RNAscope ISH combined with IHC in thick free-floating rat brain tissue sections. The dopaminergic nigrostriatal system of rats was unilaterally lesioned using 6-OHDA (a dopaminergic neurotoxin) delivered to the striatum. One week later the brains were collected and tissue was processed for Sprr1a ISH (brown) and tyrosine hydroxylase (TH) IHC (blue). A) Low magnification images clearly depict the loss of dopaminergic (i.e. TH+) neurons in the lesioned (right side) substantia nigra compared to the unlesioned hemisphere (left side). Note the presence of substantial Sprr1a ISH signal in the lesioned hemisphere, and the lack of signal in the unlesioned hemisphere. B and C) High magnification images of a 20μm thick section show clear dual labeling with ISH and IHC in the lesioned side (C), but not the unlesioned side (B). D and E) Images from a 40μm thick section show similar results. F-H) Neurons in the lesioned hemisphere exhibited little to no ISH with strong IHC (F), intermediate ISH and IHC (G), and strong ISH with little to no IHC (H). Scale bars: A = 500μm, B-E = 40μm and F-H = 20μm.

We used RNAscope ISH to detect Sprr1a and TH to label dopamine neurons in the substantia nigra. In the example shown in **Fig. 2H and 2I** brown puncta labeling Sprr1a transcripts are readily apparent. The combination of detecting Sprr1a mRNA with DAB (brown) and detecting TH IHC with Vector SG (blue-grey) provides good contrast between the two signals (**Fig. 2A, 2C, 2E, 2H and 2I**). The specificity of RNAscope labeling is apparent when comparing the lesioned hemisphere to the unlesioned hemisphere because Sprr1a is significantly induced following the lesion (**Fig. 2B and 2D** compared to 2**C and 2E**). Accordingly, little to no signal is present in the unlesioned side (**Fig. 2A, 2B and 2D**). In contrast, numerous neurons contain signal ranging from sparse puncta to nearly the entire cytoplasm filled with puncta are present in the lesioned hemisphere (**Fig. 2F-2H**). The dual ISH/IHC approach in both 20μm (**Fig. 2B and 2C**) and 40μm (**Fig. 2D and 2E**) thick sections works well, but 20μm thick sections are preferred (especially for measurements of ISH signal). These data clearly demonstrate that the combination of ISH and IHC is easily accomplished in thick free-floating brain sections.

**Fig 3:**
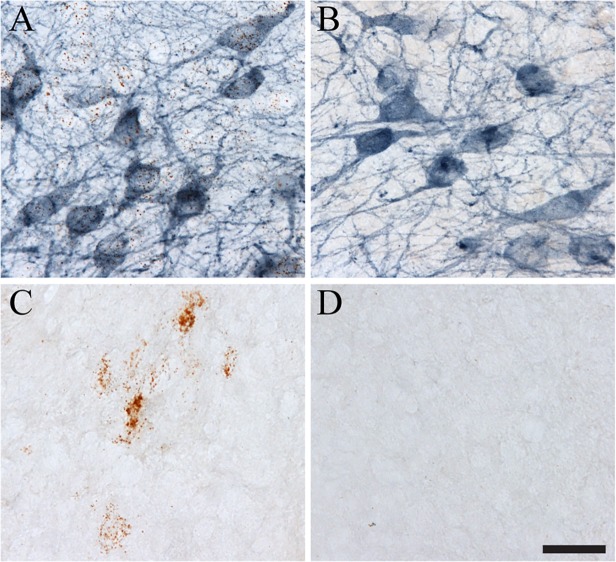
Control sections for ISH-IHC dual labeling in thick free-floating sections. A) Tissues were labeled with a rat positive control probe (i.e. rat peptidylprolyl isomerase B gene) provided by ACD. Clear ISH signal and IHC staining were observed. This probe acts as a positive control because it is present ubiquitously throughout rat tissue, albeit at modest levels in the cells depicted. B) Tissues were labeled with a negative control probe (i.e. *Bacillus subtilis* dihydrodipicolinate reductase gene) provided by ACD. Very infrequent ISH puncta are present, while strong IHC is clear. This probe was used as a negative control because it is a bacterial transcript not present in rat tissue. C) Tissue was labeled with Sprr1a ISH, but the primary TH antibody was excluded. As expected, strong Sprr1a ISH is apparent in the SN of the lesioned hemisphere (i.e. brown ISH puncta), but no TH IHC is present (i.e. lack of blue staining). D) To ensure the signals were dependent upon the Sprr1a ISH probe and TH primary antibody we omitted the Sprr1a probe and the TH primary antibody. As expected, this results in no signal for ISH (i.e. no brown puncta) or IHC (i.e. no blue staining). Scale bar: A-D = 50μm.

A number of controls were performed to assess the specificity of RNAscope and the combination of ISH with IHC. First, sections were stained with a probe against the rat peptidylprolyl isomerase B gene as a positive control probe that is present ubiquitously in rat tissue (ACD, 313921; **Fig. 3A**). This probe is provided by ACD and produced ISH signal in the rat brain sections, albeit at modest levels. The tissue was processed for ISH using this probe and then for TH IHC to identify DA neurons. As expected, the positive ISH control produced signal (i.e. brown puncta) in cells, and DA neurons were labeled blue. It is worth noting that a number of experimental probes used in this work are suitable positive control probes as well (e.g. rat βIII-tubulin and rat β-actin, see below). An RNAscope probe against the *Bacillus subtilis* dihydrodipicolinate reductase gene was used as a negative ISH control because this probe is against a bacterial gene not present in rat tissue (ACD, 310043; **Fig. 3B**). The section was processed for ISH using this probe and then labeled with TH IHC to identify DA neurons in the SN. As expected, no ISH signal (i.e. no brown puncta) was detected in the section, and DA neurons were labeled blue. In the ISH-IHC dual staining procedure, the ISH signal is developed first and then the IHC signal is developed. Thus, we processed tissue sections through the Sprr1a ISH steps normally and then omitted the primary TH antibody to ensure that the detection of TH IHC in this procedure did not produce unwanted effects on the ISH signal or erroneous signals (**Fig. 3C**). As expected, this produced clear brown punctate ISH signal, and no detectable blue staining. Finally, the Sprr1a ISH probe and TH primary antibody were omitted during the procedure. This ensures that ISH and IHC signals were dependent upon the ISH probe and IHC primary antibody and not due to non-specific artifact signal from other steps in the procedure. As expected, there was no ISH signal (i.e. no brown puncta) and no IHC signal (i.e. no blue cells) when the Sprr1a probe and TH primary antibody were omitted (**Fig. 3D**). Together, all of the controls confirm the validity of the signals observed in the ISH-IHC dual staining procedure.

**Fig 4:**
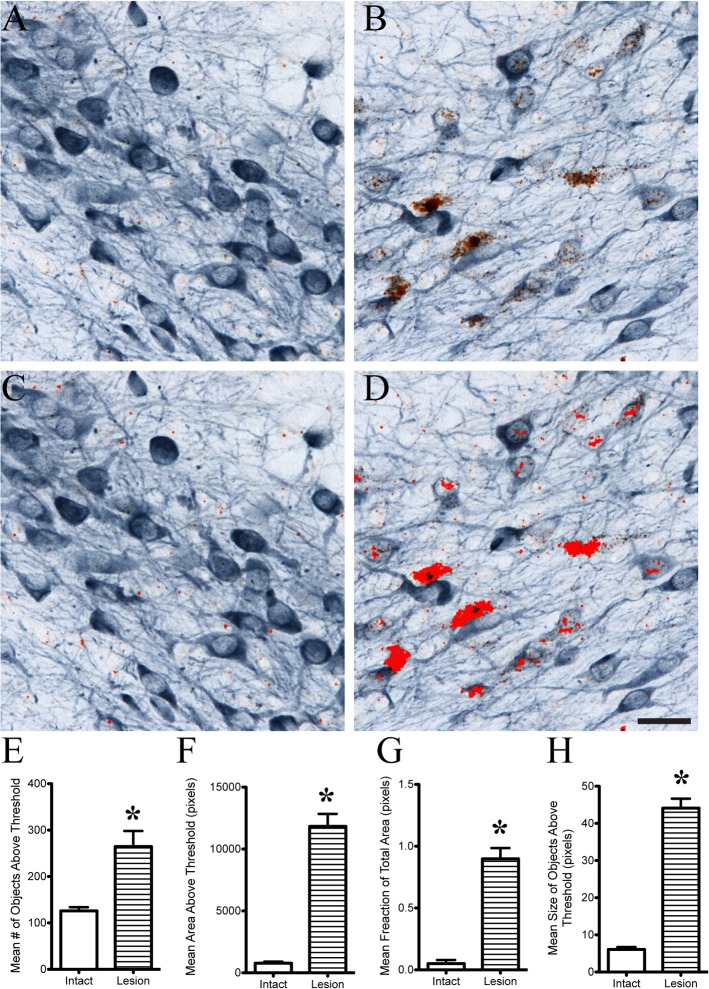
Semi-quantitative regional analysis of ISH signal in dual labeled (ISH-IHC) thick tissue sections. A-D) Images were processed using ImageJ software to set the HSB index and hue was 0–128, brightness was 0–255 and saturation was 0–141. The pixels within the threshold limit are indicated by the bright red overlay. These setting allowed a distinction with fair accuracy between the Sprr1a ISH signal (brown) and the TH IHC signal (blue). The red areas in C and D are the pixels analyzed after the thresholds were set. E-H) After applying the threshold settings, image analysis was used to measure the average number of objects (E), mean area of pixels (F), mean fraction of the total area (G), and mean size of objects (H) (*p<0.05 compared to intact). With each measurement, the lesioned side is significantly greater than the unlesioned hemisphere. Scale bar: A-D = 40μm.

**Fig 5:**
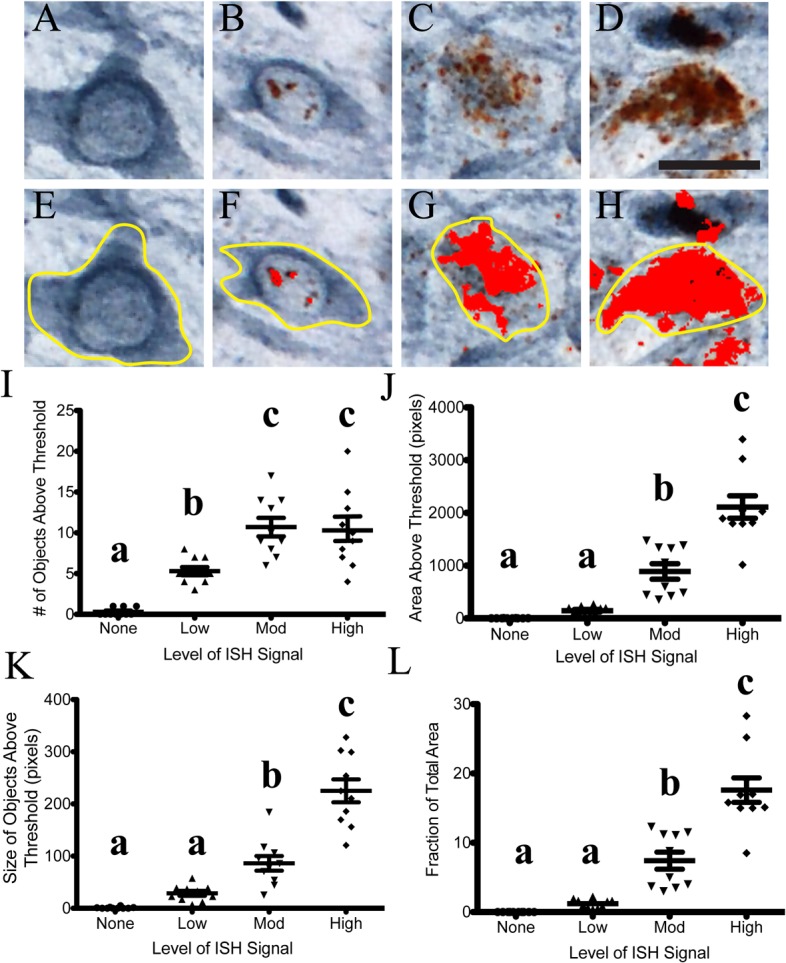
Semi-quantitative single-cell analysis of ISH signal in dual labeled (ISH-IHC) thick tissue sections. A-D) Four groups of TH+ neurons were found with varying amounts of ISH signal in the lesioned hemisphere (using same threshold settings as in **Fig. 3**). Individual neurons contained no/very little (A and E), low (B and F), moderate (C and G), and high (D and H) levels of ISH signal. The red areas in E-H are the pixels analyzed after the thresholds were set. I-L) These groups were distinguishable using the mean object number (I), mean area (J), size of the objects (K) and the fraction of the total area (L). The object number measurement detected significant differences between all four groups (p<0.05), while all other measurements identified differences between the low, moderate and high level of ISH groups. Scale bar: A-H = 20μm.

Semi-quantitative analysis of the ISH signal is possible in ISH/IHC stained tissue sections. The use of hue, saturation and brightness (HSB) color thresholding allows one to distinguish the two signals with a surprising degree of specificity, albeit not an absolutely specific method (i.e. some ISH puncta are not analyzed). Using ImageJ software, the thresholds were set to 0–128 for hue, 0–255 for saturation and 0–141 for brightness to distinguish the DAB ISH signal from the Vector SG IHC signal. This method was used to successfully measure the level of ISH signal in regional analyses using the entire image area (**Fig. 4**). Analyses of the pixels identified by the threshold settings (number of objects, area, fraction of the total area and size of objects) confirm the significant increase of Sprr1a in the lesioned hemisphere compared to the intact hemisphere (**Fig. 4C-4F**). Moreover, these settings allowed us to distinguish 4 levels of ISH signal as demonstrated by measurements made in individual cells with varying degrees of ISH signal (**Fig. 5A-5D**). Here, the same thresholding parameters were applied to individual neurons. Again, number of objects, area, fraction of the total area and size of objects can identify different populations of neurons with varying amounts of ISH signal (**Fig. 5E-5H**). Ultimately, the settings for thresholding need to be determined empirically with each stain. The goal for quantitation should be to include as much ISH signal, while excluding as much IHC signal as possible. Collectively, these data demonstrate that ISH/IHC in thick free-floating brain sections can be analyzed using semi-quantitative analyses.

### Versatility of ISH and IHC method

**Fig 6:**
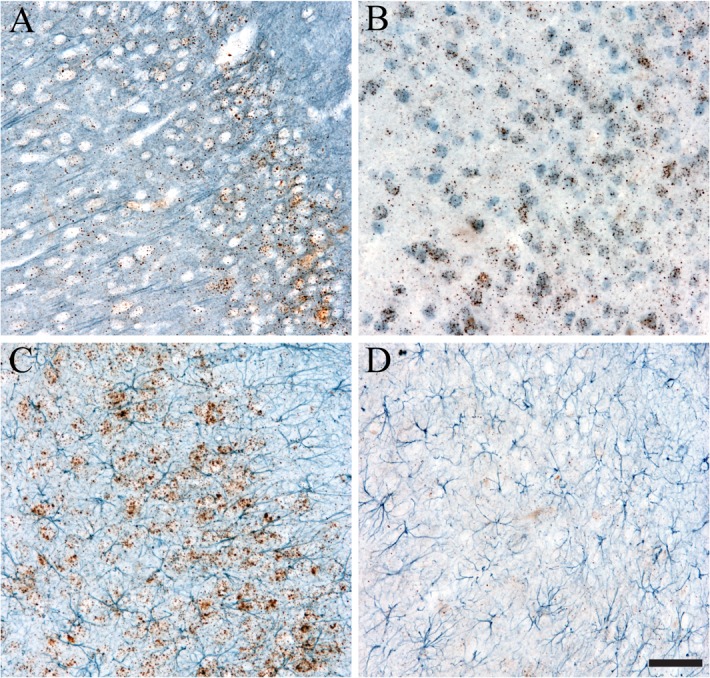
The versatility of ISH-IHC dual staining method. A number of combinations with different ISH and IHC stains were performed. A) Rat βIII-tubulin ISH (brown) and βIII-tubulin IHC (blue) were combined to label the mRNA and protein of the same target. B) Rat βIII-tubulin ISH was combined with IHC for NeuN, a common neuron-specific marker. C) Rat β-actin ISH was combined with IHC for GFAP, a common astrocyte-specific marker. Both B and C confirm that cell type-specific markers are compatible with this ISH-IHC dual staining method. D) Sprr1a ISH was combined with GFAP IHC to demonstrate that the same ISH marker can be combined with multiple IHC markers (i.e. TH in Figs. 2–5 and GFAP here). All images were taken in the area of the retrosplenial cortex. Scale bar: A-D = 50μm.

Next, we tested robustness of this method by determining if it could be applied to a number of ISH-IHC combinations. The successful combination of ISH for βIII-tubulin with βIII-tubulin IHC confirms that the same target can be used for each method (**Fig. 6A**). The ISH for βIII-tubulin was combined with NeuN IHC as a neuron-specific marker (**Fig. 6B**), while β-actin ISH was combined with GFAP IHC as an astrocyte-specific marker (**Fig. 6C**) to establish whether cell type-specific markers could be used. Finally, Sprr1a ISH was combined with GFAP IHC to show that the same ISH probe can be used with multiple IHC labels (**Fig. 6D**) (also combined with TH). Together, these data demonstrate that multiple ISH probes and multiple IHC antibodies (including those to neurons and glial cells) can successfully be applied using this method. Importantly, the success and utility of any ISH probe is not influenced by the IHC stain it is combined with and as such any ISH probe and any IHC stain that is deemed to work independently should work as well in combination.

### Combining ISH and IHC in Primary Neuron Cultures

**Fig 7:**
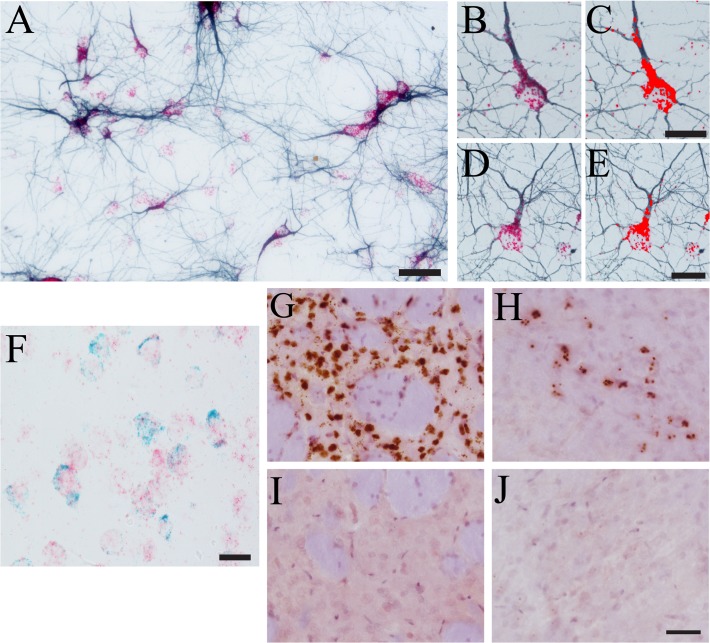
RNAscope ISH-IHC in primary neuron cultures, dual RNAscope ISH detection, and detection of ectopic viral vector DNA. A) Primary rat neurons were processed for tau ISH and βIII-tubulin IHC. Clear puncta are present for the tau ISH signal (red), while neuronal cell bodies and processes are clearly labeled with tubulin IHC (blue). B-E) Color thresholding (using the YUV index) allows clear separation of ISH signal (red puncta) from the IHC signal (blue staining) in primary neurons. Here, the YUV index was used and Y was 0–135, U was 0–255 and V was 135–255. The pixels within the threshold limits are shown by the bright red overlay. F) Dual ISH for tau (red) and βIII-tubulin (blue/green) was performed on 40μm thick rat brain sections (cortex depicted). G-J) Rats were unilaterally injected in the striatum with recombinant adeno-associated viruses (rAAV, serotype 2/5). RNAscope was used to detect rAAV DNA. Robust rAAV DNA ISH signal is detected in the injected striatum (G) compared to the uninjected hemisphere (I). ISH signal was also detected in the SN ipsilateral to the injected striatum because rAAV particles are retrogradely transported to the SN (H), no signal is detected in the contralateral SN (J). Scale bars: A = 50μm, C and E = 20μm, F = 25μm, and J = 50μm.

Our next goal was to determine whether RNAscope would be effective at labeling mRNAs in primary neurons grown in culture, and whether ISH could be combined with IHC in culture. We used primary rat hippocampal neurons and tested RNAscope using a tau probe (a channel 2 probe—red chromogen detection), and the cells were counterstained using IHC for βIII-tubulin (blue chromogen). The signal for tau ISH was readily apparent (red puncta) and the tubulin stain worked very well to identify the processes of neurons (blue; **Fig. 7A-7E**). This also demonstrates that channel 2 probes can be combined with the VectorSG blue IHC substrate. Together, these data are the first to demonstrate that RNAscope can be used in primary neuron cultures and that it can be used in conjunction with IHC in these cells.

Importantly, the signals from the channel 2 probes (i.e. red puncta) are easily separated from the blue IHC staining by color thresholding. Two illustrative examples of neurons dual labeled with tau ISH (red puncta) and βIII-tubulin IHC (blue staining) are depicted in **Fig. 7B-7E**. Application of the color thresholding method allows for remarkable separation of the red puncta from the blue colored IHC staining, where the bright red pixels indicate the pixels within the set threshold limits (**Fig. 7C and 7E**). This allows semi-quantitative analyses as described for the tissue sections (above). However, it is important to note that a different color threshold index is used in this example because the chromogen combination is red and blue. The use of a channel 1 probe (i.e. brown puncta) and the blue IHC chromogen should allow analysis with the same method described above for the tissue. Nonetheless, these data clearly indicate the usefulness of this dual labeling procedure in primary neuron cultures and show the ability to semi-quantitatively measure the ISH signal produced.

### Double-label RNAscope in thick free-floating brain sections

Next, we were interested in developing the ability to co-localize two RNAscope labels in thick free-floating tissue sections. Here, we used tau ISH combined with βIII-tubulin ISH in rat brain sections. As expected, the co-localization of each label was evident because the labeling produced clearly distinguishable puncta. Neurons were positive for both tau and tubulin (**Fig. 7F**). Thus, the double-label RNAscope ISH procedure works well in 40μm and 20μm (data not shown) thick free-floating tissue sections.

### Ectopic DNA detection with RNAscope technology in free-floating brain sections

Often, neuroscience research relies on the use of gene therapy approaches to deliver therapeutic agents (e.g. trophic factors) or in the generation of animal models. Here, we used rAAVs injected into the striatum of rats as a proof of principle that RNAscope can be used to detect ectopic DNA molecules (**Fig. 7G-7J**). The rAAV-injected striatum contains robust ISH signal (**Fig. 7G**), while the uninjected striatum is devoid of ISH signal (**Fig. 7I**). It is noteworthy that the retrograde transport of rAAVs along the nigrostriatal pathway is readily detectable as ISH signal in the substantia nigra of the injected hemisphere (**Fig. 7H**); no signal is seen in the uninjected hemisphere (**Fig. 7J**). To our knowledge this is the first demonstration of a method for identifying neurons transduced with rAAVs using ISH for the viral genome particles.

### General Considerations, Expected Results, and Troubleshooting

The RNAscope signal should produce clear and intense puncta as the labeling of single transcripts (see **Fig. 1**) [9,11]. When a robust signal is detected the puncta can fill a large portion of the cytoplasm (see **Figs. 2 and 5**), which can obscure individual puncta. The IHC signal from Vector SG should produce a blue-grey colored staining that follows a pattern consistent with known patterns for the antigen being detected. The signal for both stains should penetrate the entire z-plane through the tissue section. This technique can be applied to archival tissue as we have successfully used tissue sections that have been stored in cryoprotectant for multiple years (data not shown).

When performing the double label ISH-IHC stain, the RNAscope puncta should not be blue-grey. If such a result is obtained this may indicate that the RNAscope signal was not fully developed and/or the second peroxidase-quenching step was insufficient. Alternatively, other peroxidase substrates (e.g. NovaRed) or an alkaline phosphatase substrate can be used for the IHC detection, but phosphatase substrates can produce high background in brain tissue due to high levels of endogenous phosphatase activity. It is preferable to use 20μm thick sections because this potential background issue is significantly mitigated (often eliminated) and this thickness is more amenable to semi-quantitative analyses because there is less out-of-focus tissue that can interfere with detection.

Occasionally, diffuse coloration of the tissue occurs from the DAB reaction if the tissue becomes ruffled or starts to come off the slide during the RNAscope procedure. The best way to reduce this is to make sure care is taken to flatten the tissue onto the slide and “bake” the tissue onto the slide at 60°C as described in the methods. The diffuse background signal does not resemble positive RNAscope signal (i.e. strongly stained discrete puncta), which makes it easily distinguishable from positive RNA staining. Occasionally, perivascular background signal can occur as well, which may be due to inadequate peroxidase quenching or poor transcardial perfusion of the animal.

## Discussion

*In situ* hybridization has been used for almost 50 years with the explicit purpose of identifying specific genes (i.e. DNA or mRNA transcripts) in specific cell populations. Indeed, large ISH databases exist that can provide information (some provide semi-quantitative data) on numerous genes in various tissue types, such as the Allen Brain Atlas, Eurexpress, the Edinburgh Mouse Atlas Gene Expression Database, and the GenePaint database. Often, the traditional ISH methods were not sensitive enough to localize single transcripts in specific individual cells in tissue sections. With the development of ISH methods that utilized chromogenic enzyme substrates to detect ISH signals, or more recent fluorescence-based ISH methods the localization to single cells was possible [2–6,8]. However, single transcript sensitivity, processing thick free-floating sections and combining ISH with IHC were not readily performed, or simply were not feasible with traditional ISH methods.

The commercially available RNAscope technology can provide single transcript sensitivity [9]. Here, we describe the use of this technique in a number of formats. We outline a novel method for combining this form of ISH with IHC in thick free-floating tissue sections from the rodent brain. The versatility of this method is highlighted by our ability to combine ISH and IHC probes for the same target, ISH probes with common neuron- and glial-specific markers, and using different IHC probes with the same ISH probe. We provide the first demonstration that this combined ISH-IHC approach is readily applicable in primary neurons grown in culture. We show that RNAscope provides a means to perform double-label ISH signal detection in thick brain sections. Finally, we demonstrate that this technology can be used to detect ectopic viral genome DNA. ISH methods have been used in the virology field for many years [20,21], but to our knowledge this is the first use of ISH to detect viral DNA genomes from exogenous rAAVs used to transduce rodent brain tissue. This level of versatility and ease of use make this approach very attractive to laboratories currently utilizing other ISH techniques, as well as ISH novices.

One important aspect of the ISH technique described here is the relative ease with which the signal can be measured. The ISH signal can be semi-quantitatively analyzed to assess the relative amount of signal using image thresholding techniques within individual neurons or within regional areas, and within primary neurons. Moreover, we show that both combinations of channel 1 probes (brown puncta) with blue IHC and channel 2 probes (red puncta) with blue IHC can be analyzed using relatively simple color thresholding techniques. Importantly, these data are not truly quantitative because it is difficult to accurately count individual puncta. If the signal is strong enough, the puncta can “merge” into indistinguishable clusters of ISH-derived staining. The presence of the IHC interferes with measuring all of the ISH puncta, but the IHC is required if identifying specific, individual neurons is the goal. If the IHC is omitted, one may be able to more accurately quantify the absolute amount of ISH signal and thereby approach a more quantitative measurement, but the ability to localize the ISH within a specific cell type is not possible. Thus, the data obtained with this method allow comparisons of “relative” differences in ISH signal within specific cells or regions.

Here, we used a rodent model of unilateral dopamine neuron degeneration (i.e. the 6-OHDA rat model [15]) to demonstrate that the ISH signal can be localized to specific cells (i.e. nigral dopamine neurons identified with TH IHC) and can be semi-quantitatively measured in thick tissue sections when ISH and IHC are combined. Specifically, significant upregulation of Sprr1a occurred in nigral dopamine neurons after 6-OHDA administration in the striatum. Sprr1a is an injury-induced gene in neurons [13,14], a characteristic that is highlighted by the lack of Sprr1a ISH signal in the unlesioned hemisphere of the animals. Measurements of Sprr1a ISH provided a distinction between individual cells with no, low, moderate and high levels of Sprr1a expression in the nigra. Moreover, regional analyses of the intact and lesioned nigra detected a significant increase in signal within the lesioned hemisphere compared to the intact hemisphere. These experiments confirm the usefulness of semi-quantitatively analyzing the relative level of gene expression changes using these techniques.

The utilization of this highly sensitive method for oligonucleotide detection in thick free-floating sections significantly adds to the capability of researchers in a number of fields. This technique allows ISH studies using archival tissue resources that are typically intended almost exclusively for IHC. Moreover, the combination of sensitive ISH with IHC will allow for the specific localization of gene changes in specific cell populations. RNAscope ISH also provides a useful technique to identify specific cell populations when antibodies against the protein of interest are not available. Finally, the semi-quantitative nature of the staining produced by RNAscope when combined with IHC adds an extra level of utility to this technique that would be well complemented by additional quantitative methods (e.g. RNAscope without IHC, or quantitative real time PCR using fresh tissue [22,23]). Altogether, the use of RNAscope and IHC is an extremely versatile and useful technique that can easily be incorporated into the toolbox of any laboratory for addressing specific scientific questions that are not readily answerable with other approaches.

## Supporting Information

S1 Protocol A detailed laboratory protocol. Outlines the entire process for combining RNAscope with IHC, including product numbers and recipes for reagents.
(DOCX)

S1 Fig Flattened paintbrush bristles used to smooth tissue sections onto microscope slides. Care must be taken to ensure the tissue is well adhered and completely flat on the microscope slide. After the mounting the section onto the slide allow it air-dry for a 30–60s. Then wet the bristles of a small paintbrush (A) and squeeze the bristles between paper towel (B) to flatten and fan the bristles (C). Then very gently flatten the tissue section with the tips of the bristles (arrows in C). The bristles should also wick away remaining moisture as the section is flattened. Ensure the tissue section is flat before processing through the remainder of the protocol.
(TIF)
