# Supplementary material for: A Method for Combining RNAscope In Situ Hybridization with Immunohistochemistry in Thick Free-Floating Brain Sections and Primary Neuronal Cultures
Source: PLoS One. 2015 Mar 20;10(3):e0120120. doi: 10.1371/journal.pone.0120120 (PMC4368734; doi:10.1371/journal.pone.0120120)
Supplement: S1 Protocol — Outlines the entire process for combining RNAscope with IHC, including product numbers and recipes for reagents. (DOCX) [file pone.0120120.s001.docx]

**Combined RNAscope *in situ* hybridization and immunohistochemistry protocol**

*20µm free-floating tissue section protocol:*

1. Remove sections from cryoprotectant
2. Wash four times in TBS for 10 min each
3. Incubate in Pretreatment 1 at RT until bubbling stops (45-60 min)
4. Wash four times in 0.5xTBS for 1min each
5. Mount one section per slide
6. Flatten on the slide
   1. No folds/overlaps in the section
   2. After ~30-60s, use the tips of the flattened paintbrush bristles to carefully flatten tissue (**Figure S1**)
7. Once mounted, dry sections at either RT or 60°C
8. Once dry remove residual salts by rapidly dipping in H_2_O
9. Dry overnight at 60°C on a slide warmer
10. The following day, incubate in a boiling (99-104°C) solution of 1X Pretreatment 2 for 5-10 min
11. Wash in H_2_O two times for 1min each
12. Dry at RT
13. Dip in 100% EtOH and air-dried before creating a hydrophobic barrier
14. Proceed to Pretreatment 3 (see below).

*40µm free-floating section protocol*:

1. Remove sections from cryoprotectant
2. Wash four times in TBS for 10 min each
3. Incubate in Pretreatment 1 at RT until bubbling stops (45-60 min)
4. Wash four times in TBS for 1 min each.
5. Incubate free-floating sections in 1X Pretreatment 2 pre-heated to 99-100°C for 10 min
6. I*mmediately* remove from Pretreatment 2 and place in 0.5xTBS
7. Mount directly onto slides (one section per slide) as above (i.e. steps 5-14).
8. Move to pretreatment 3.

*Pretreatment 3:* (20μm and 40μm sections are processed identically)

1. Incubate in Pretreatment 3 (~2-3 drops/per section) at 40°C for 15 min
2. Wash four times in H_2_O for 1min each

*Probe Incubation for Single-Plex Probes:*

1. Incubate in desired probe (~2-3 drops/section) for 2 hours at 40°C.
2. Wash four times in 1x wash buffer for 1 min each.

*Amp 1-6 for Single-Plex Probes:*

1. Incubate in Amp1 solution for 30 min at 40°C
2. Wash four times in wash buffer for 1 min each
3. Incubate in Amp2 solution for 15 min at 40°C
4. Wash four times in wash buffer for 1 min each
5. Incubate in Amp3 solution for 30 min at 40°C
6. Wash four times in wash buffer for 1 min each
7. Incubate in Amp4 solution for 15 min at 40°C
8. Wash four times in wash buffer for 1 min each
9. Incubate in Amp5 solution for 30min at RT
10. Wash four times in wash buffer for 1 min each
11. Incubate in Amp6 solution for 15 min at RT
12. Wash four times in wash buffer for 1 min each.

*Detection for Single-Plex Probes:*

1. Mix equal parts of diaminobenzidine (DAB)-A solution to DAB-B solution
2. Incubate sections for 10 min at RT
3. Wash in H_2_O two times for 2 min
4. Counterstain sections as desired or move to immunohistochemistry as described below

*RNAscope in cultured primary neurons*:

1. Incubate in Pretreatment 1 solution at RT for 10 min
2. Wash two times in TBS
3. Incubate in Pretreatment 3 (diluted 1:15 in TBS) at RT for 5 min
4. Wash two times with TBS
5. Incubate in the probe solution as described above
6. All of the remaining steps were the same as described above

*Immunohistochemistry (IHC) after ISH detection:*

1. Immediately after ISH detection, smooth sections using a flattened paintbrush
2. Dry at 60°C for several minutes before proceeding
   1. Alternatively, dry at 60°C overnight
3. Once dry, reapply hydrophobic barrier
4. Incubate in 1% H_2_O_2_ for 10 min at RT
5. Wash in TBS four times for 4 min each
6. Incubate in blocking buffer (10% serum from the host species of secondary antibody) for 30 min at RT
7. Incubate in primary antibody at RT overnight
8. The following day, wash four times in TBS for 4 min each
9. Incubate in biotinylated secondary antibody diluted in 2% serum for 2 hours at RT
10. Wash four times in TBS for 4 min each
11. Incubate in avidin-biotin complex solutions for 2 hours at RT
12. Wash four times in TBS for 4 min each
13. Detect IHC using the VectorSG peroxidase substrate (~5-8 min)
14. Wash in TBS four times for 1 min each
15. Dehydrate through graded EtOHs (i.e. 50%, 70%, 95%, and 100%) for 1 min each
16. Clear in xylenes for 2 min
17. Coverslip using Cytoseal-60 mounting media.
