# Supplementary figures and images for: A Method for Combining RNAscope In Situ Hybridization with Immunohistochemistry in Thick Free-Floating Brain Sections and Primary Neuronal Cultures
Source: PLoS One. 2015 Mar 20;10(3):e0120120. doi: 10.1371/journal.pone.0120120 (PMC4368734; doi:10.1371/journal.pone.0120120)

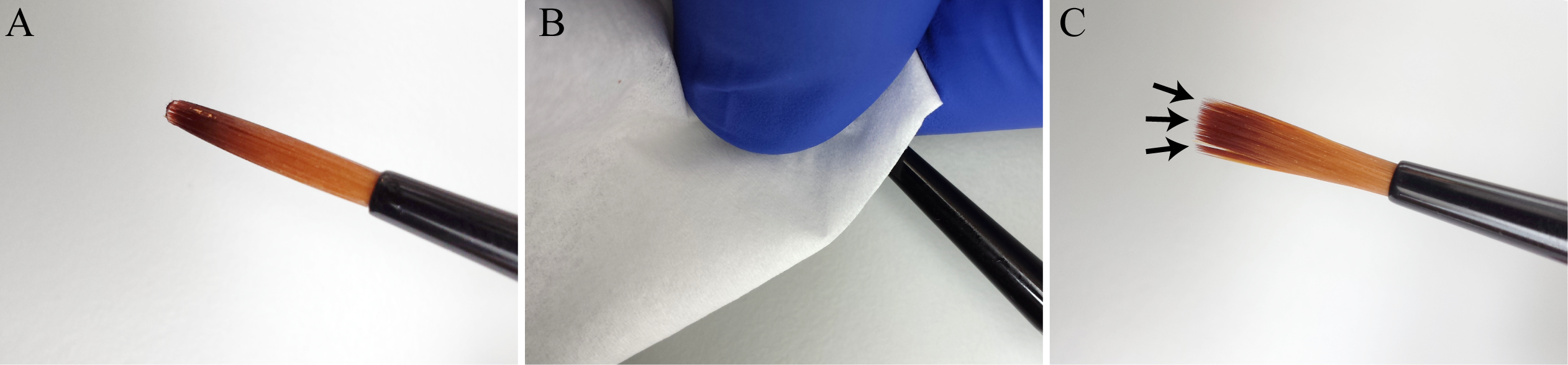

Supplement: S1 Fig — Care must be taken to ensure the tissue is well adhered and completely flat on the microscope slide. After the mounting the section onto the slide allow it air-dry for a 30–60s. Then wet the bristles of a small paintbrush (A) and squeeze the bristles between paper towel (B) to flatten and fan the bristles (C). Then very gently flatten the tissue section with the tips of the bristles (arrows in C). The bristles should also wick away remaining moisture as the section is flattened. Ensure the tissue section is flat before processing through the remainder of the protocol. (TIF) [file pone.0120120.s002.tif]
